# Dysregulated Gab1 signalling in triple negative breast cancer

**DOI:** 10.1186/s12964-024-01542-9

**Published:** 2024-03-06

**Authors:** Hannes Bongartz, Nora Mehwald, Elena A. Seiß, Tim Schumertl, Norbert Naß, Anna Dittrich

**Affiliations:** 1 Institute of Biology, Department of Systems Biology, Otto-von-Guericke University, Universitätsplatz 2, Magdeburg, 39106 Germany; 2 Department of Pathology, Brandenburg Medical School Theodor Fontane, University Hospital Brandenburg / Havel, Hochstraße 29, Brandenburg, 14770 Germany; 3 Center for Dynamic Systems: Systems Engineering (CDS), Otto‐von‐Guericke University, Universitätsplatz 2, Magdeburg, 39106 Germany; 4 Magdeburg Center for Systems Biology (MACS), Otto‐von‐Guericke University, Universitätsplatz 2, Magdeburg, 39106 Germany; 5 Present address: Laboratory of Immune System Biology, National Institute of Allergy and Infectious Diseases, National Institutes of Health, 9000 Rockville Pike, Bethesda, MD 20892 USA; 6 Present address: Institute of Clinical Biochemistry, Hannover Medical School, Carl-Neuberg-Straße 1, Hannover, 30625 Germany

**Keywords:** Triple-negative breast cancer, MDA-MB-468, Gab1, PI3K, MAPK, EGFR, Acquired resistance

## Abstract

**Background:**

Breast cancer is the most common cancer in women worldwide. Triple-negative breast cancer (TNBC) is especially aggressive and associated with high metastasis. The aetiology of TNBC is heterogeneous and characterised by multiple different mutations that amongst others cause constitutive and dysregulated MAPK and PI3K signalling. Additionally, in more than 50% of TNBC patients, the epidermal growth factor receptor (EGFR) is overexpressed and constitutively active. The multi-site docking protein Grb2-associated binder 1 (Gab1) is a central signalling hub that connects MAPK and PI3K signalling.

**Methods:**

Expression and activation of members of the Gab1/PI3K/MAPK signalling network were assessed in cells from different breast cancer subtypes. Influence of short- and long-term inhibition of EGFR, MAPK and PI3K on the activation of the Gab1/PI3K/MAPK signalling network as well as on cell viability, proliferation and migration was determined. Additionally, cellular localisation of Gab1 and Gab1 variants in naive cells and cells treated with the above-mentioned inhibitors was investigated.

**Results:**

We show that, activation of the Gab1/PI3K/MAPK signalling network is heterogeneous between different breast cancer subtypes. Gab1 phosphorylation and plasma membrane recruitment of Gab1 are dysregulated in the EGFR^high^ TNBC cell line MDA-MB-468. While the Gab1/MAPK/PI3K signalling network follows canonical Gab1 signalling in naive MDA-MB-468 cells, Gab1 signalling is changed in cells that acquired resistance towards MAPK and PI3K inhibition. In resistant cells, Gab1 is not located at the plasma membrane despite strong activation of PI3K and MAPK. Furthermore, Gab1 tyrosine phosphorylation is uncoupled from plasma membrane recruitment.

**Conclusion:**

Our study indicates that Gab1 signalling changes fundamentally during the acquisition of resistance to pharmacological inhibitors. Given the molecular heterogeneity between breast cancer subtypes, the detailed understanding of dysregulated and aberrant signalling is an absolute necessity in order to develop personalised therapies for patients with TNBC.

## Abstract (plain language summary)

Breast cancer is very diverse among different patients. Understanding these differences is important for specific and successful treatment of breast cancer patients. About 15% of breast cancer patients have a very severe form of breast cancer called triple negative breast cancer. So far, no specific treatment for these patients exists. Triple-negative breast cancer cells divide without external stimuli as intracellular signalling is constitutively activated in these cells. We show that, in a specific type of triple negative breast cancer, an intracellular signalling network called Gab1/MAPK/PI3K signalling is disturbed. In these breast cancer cells, the Gab1/MAPK/PI3K network is initiated by hyperactive epidermal growth factor receptor (EGFR). In naive untreated breast cancer cells, the EGFR-induced Gab1/MAPK/PI3K network follows the rules described for healthy cells. However, when the cells acquire resistance to pharmacological inhibition of this network, substantial changes in this network happen. This study is the first showing that Gab1 signalling fundamentally changes during resistance development. Understanding the underlying molecular changes during cancer progression is fundamental for future development of personalised therapies for patients with triple negative breast cancer.

## Background

Breast cancer (BC) is the most commonly diagnosed cancer in women worldwide with an estimated 2.3 million new cases per year [1]. The overall survival of BC patients in advanced stages is still very poor [2]. BC development and progression are caused by various changes in healthy cells of breast tissue [3]. Heterogeneity between different BC subtypes complicates diagnosis and calls for individual treatment options. Unfortunately, the diverse interconnected molecular pathways that result in proliferation and metastasis of BC cells are not completely decoded yet. Therefore, elucidating the molecular mechanisms accounting for disease progression in defined subtypes of BC is a necessity for personalised therapy.

Triple-negative breast cancer (TNBC) accounts for 15 to 20% of all breast cancers and is characterised by high rates of metastasis and recurrence, and hence poor prognosis [4, 5]. So far, conventional cytotoxic chemotherapy and surgery are the mainstay therapy for TNBC patients [6]. To develop more specific and even personalised treatment options, it is essential to understand the mechanisms underlying dysregulated signalling pathways in TNBC [7]. TNBC cells are defined by the lack of estrogen receptor (ER), progesterone receptor (PR) and epidermal growth factor receptor 2 (ErbB-2, Her2) expression. Additional changes in TNBC cells delineate at least six subgroups of TNBC [8]. In a minimum of 50% of patients with TNBC the expression of the epidermal growth factor receptor (EGFR, ErbB-1) is increased. Increased EGFR expression serves as an additional negative prognostic marker for BC patients [9]. Overexpression of EGFR often results in ligand-independent constitutive receptor activation, which is likely caused by greatly increased ligand-independent dimerisation and intrinsic activity of the kinase domain of the EGFR [10, 11]. This constitutive activity is associated with an increased resistance to chemotherapy and augmented risk of metastasis [9, 12, 13]. EGFR activity accounts for aberrant mitogen activated protein kinase (MAPK) pathway activation [14] that is associated with growth and survival of TNBC cells [15]. Based on these observations, targeting of MAPK signalling was considered a promising therapeutic approach to treat patients with an EGFR^high^ TNBC subtype (for review, see [16]). Unfortunately, TNBC cells rapidly adapt to targeted inhibition of MAPK signalling and develop resistance (for review, see [17]). Notably, the underlying resistance mechanisms are heterogeneous between different BC subtypes and often remain elusive.

Beside constitutive activation of MAPK signalling, mutations in genes coding for proteins involved in phosphatidyl-inositol-3-kinase (PI3K) signalling are a second hallmark of dysregulated signalling in TNBC [18]. Initial clinical trials to treat TNBC with different PI3K inhibitors were however, challenged by high toxicity and only modest anti-tumour effects (for review, see [19]). Notably, inactivating mutations in phosphatase and tensin homolog deleted on chromosome 10 (PTEN), which is one of the main negative regulators of PI3K signalling, are found in several TNBC cell lines [20]. MAPK and PI3K signalling pathways are closely interconnected by complex cross-talk scenarios. It is thus tempting to believe that key molecules involved in the cross-talk between MAPK and PI3K signalling are central hubs in shaping cancer-associated signalling in TNBC.

The multi-site docking protein Grb2-associated binder 1 (Gab1) is known to regulate and interconnect MAPK and PI3K signalling (for review, see [21, 22]). Gab1 contains binding sites for the regulatory subunit p85 of PI3K, the SH2 domain containing phosphatase 2 (SHP2), the Ras-GTPase activating protein (RasGAP), and growth factor receptor-bound protein 2 (Grb2) (for review, see [22]). The orchestrated binding of these different MAPK- and PI3K-modulating proteins to Gab1 is tightly regulated by recruitment of Gab1 to the plasma membrane and phosphorylation of Gab1. Membrane recruitment of human Gab1 is initiated by MAPK-dependent phosphorylation of S551 (S552 in mice) in Gab1 (for review, see [21]). In the unphosphorylated state of Gab1, an epitope that contains S551 interacts with the Gab1 pleckstrin homology (PH) domain and thus blocks interaction of the PH domain with phosphatidylinositol (3,4,5)-trisphosphate (PIP3) within the plasma membrane [23]. Phosphorylation of S551 releases this intramolecular interaction and consequently allows recruitment of Gab1 to the plasma membrane [23]. Of note, phosphorylation of Gab1 at interaction sites for other proteins occurs both dependent and independent of plasma membrane recruitment and is highly reliant on upstream signalling events [24]. Although a contribution of Gab1 to oncogenic EGFR-induced signalling is known [25, 26] the exact role of Gab1 in the MAPK/PI3K signalling network in different TNBC subgroups remains elusive so far.

Gab1 mRNA and protein is overexpressed in different types of cancer such as ovarian cancer or myeloproliferative neoplasms but also in luminal and Her2 positive BC as well as in TNBC [24, 27, 28]. Gab1 expression is higher in TNBC compared to luminal or Her2 positive BC [28]. Furthermore, overexpression of Gab1 in BC cells induces epithelial-mesenchymal transition (EMT) and lymph node metastasis [28]. Interestingly, a mutation within the Gab1 PH domain has been identified in some BC patients [29]. In accordance, the sterical inhibition of the PH domain of Gab1 reduces survival of breast cancer cells in vitro [30]. These results indicate a pivotal role of Gab1-dependent signalling in the oncogenesis of breast tissue and might reveal clinical implications of Gab1 in BC therapy. Notably, most studies that describe Gab1 to be involved in progression of BC focus on analysing the expression of Gab1 but do not analyse its cellular localisation and its posttranslational modifications.

In this study, we show that Gab1 is constitutively phosphorylated at tyrosine 627 and located at the plasma membrane in the TNBC cell line MDA-MB-468. Short-term interference with constitutive Gab1 signalling by specific inhibition of either EGFR, MAPK or PI3K releases Gab1 from the membrane and reduces Gab1 tyrosine phosphorylation. However, long-term blockade of PI3K and MAPK signalling results in resistance of the cells against the applied inhibitors. In this state, Gab1 is no longer located at the plasma membrane. However, Gab1 is still highly phosphorylated at tyrosine 627. To our knowledge, this is the first time that Gab1 signalling has been shown to fundamentally change during resistance development.

## Methods

### Materials

Antibodies against tyrosine 627-phosphorylated Gab1 (corresponds to Y628 in murine Gab1, #3233), tyrosine 1068-phosphorylated EGFR (#3777), threonine 202- and tyrosine 204-phosphorylated ERK1/2 (#4370), tyrosine 705-phosphorylated STAT3 (#9131), serine 473-phosphorylated Akt (#4060), Akt (#4685), ERK1/2 (#4696), EGFR (#4267), STAT3 (#9139), vimentin (#5741), and E-cadherin (#3195) were obtained from Cell Signaling Technology (Danvers, MA, USA). The Gab1 specific antibody (#06–579) was purchased from Merck Millipore (Burlington, MA, USA). The α-tubulin (tubulin) specific antibody (#T5168) was obtained from Sigma-Aldrich (St. Louis, MO, USA).

MEK inhibitor U0126, PI3K inhibitor LY294002 (both Cell Signaling Technology) and the EGFR inhibitor Gefitinib (Cayman Chemical Company, Ann Arbor, MI, USA) were dissolved in DMSO (Carl Roth, Karlsruhe, Germany). RPMI 1640 (phenol red-free) medium and penicillin/streptomycin were obtained from Life Technologies (Carlsbad, CA, USA). Fetal calf serum (FCS) was purchased from GE Healthcare (Chicago, IL, USA).

### Cell culture

MCF7 (ATCC, #HTB-22), T-47D (ATCC, #HTB-133), SKBR3 (ATCC, #HTB-30), MDA-MB-468 (ATCC; #HTB-132), UACC-3199 (ATCC, #CRL-2983), MDA-MB-231 (ATCC, #CRM-HTB-26) and Hs 578T (ATCC, #HTB-126) cells were grown in phenol red-free RPMI 1640 medium supplemented with 10% FCS, 100 µg/mL streptomycin, and 60 µg/mL penicillin at 37 °C in a water-saturated atmosphere in the presence of 5% CO_2_. All cells were tested negative for mycoplasma contamination on a regular basis.

### Expression vectors

cDNAs of murine wild type Gab1 and Gab1 mutants were derived from pBAT-Gab1 [31] and subcloned into pd2eGFP-N1 (Clontech, Mountain View, CA, USA) to obtain C-terminally GFP-tagged Gab1 as described previously [32]. pd2eGFP-Gab1-WT encodes wild type Gab1. pd2eGFP-Gab1-ΔPH encodes a Gab1 mutant that lacks the N-terminal 119 amino acids comprising the PH domain. pd2eGFP-Gab1-S552A encodes a Gab1 mutant where alanine substitutes for serine 552. This mutant was derived from pd2eGFP-Gab1-WT via point mutagenesis [23]. Serine 552 in murine Gab1 corresponds to serine 551 in human Gab1 [33].

### Transfection

MDA-MB-468 cells were transiently transfected with pd2eGFP-N1 expression vectors encoding for wild type or mutant murine C-terminally GFP-tagged Gab1 using Lipofectamine 2000 (Life Technologies) according to the manufacturer’s instructions.

### Cell viability and proliferation assays

MDA-MB-468 cells were seeded in 6-well plates for 24 h. Subsequently, cells were treated with DMSO or inhibitors as indicated in the figures. Number of viable cells and viability were analysed using the trypan blue method-based cell counter system Vi-CELL XR (Beckman Coulter, Brea, CA, USA). The number of viable cells was determined by counting trypan blue negative cells, whereas the total cell count was obtained by counting trypan blue negative and positive cells. Viability was calculated by calculating the ratio of the number of viable cells to the total number of cells.

### Migration assay

MDA-MB-468 cells were seeded in 24-well plates for 24 h. After reaching confluency, a sterile 200 µL pipette tip was used to scratch a cross in each well. Detached cells were removed by washing with PBS. Subsequently, fresh medium containing FCS with or without inhibitors was added and cells were incubated for another 72 h. Before image acquisition, the medium was removed and the cells were washed with PBS. Then, pre-warmed fresh medium was added. To monitor the scratch closure, the scratch was imaged directly after scratching (initial wound area) and after 72 h. Images were taken using the EVOS FL imaging system (ThermoFisher Scientific, Waltham, MA, USA) with an objective providing 4x magnification. Phase contrast pictures were taken from two randomly selected fields per dish. Total area and area covered with cells were quantified using CellProfiler [34]. Relative wound recovery was calculated as ratio of the area covered with cells after 72 h and the area covered with cells initially after the scratch.

### Western blotting

Cells were lysed in RIPA lysis buffer (50 mM Tris-HCl; pH 7.4, 150 mM NaCl, 0.5% Nonidet P-40, 15% glycerol), supplemented with NaF (1 mM), Na_3_VO_4_ (1 mM), 4-(2-Aminoethyl)-benzolsulfonylfluorid (AEBSF) (0.8 µM) (Carl Roth) and 10 µg/mL of each aprotinin, pepstatin (Sigma-Aldrich), and leupeptin (MP Biochemicals, Irvine, CA, USA). Protein concentrations of the lysates were determined by Bio-Rad Protein Assay (Bio-Rad, Hercules, Ca, USA) according to the manufacturer’s instructions. Equal amounts of protein per sample were separated by SDS-PAGE and transferred to a nitrocellulose membrane. After blocking with Roti®Block (Carl Roth), membranes were incubated with specific primary antibodies (1:1000 in TBS-N; pH 10.4 (20 mM Tris-HCl; pH 7.6, 140 mM NaCl, 140 mM Nonidet P-40)) and subsequently incubated with secondary IRDye 800CW-conjugated anti-rabbit, IRDye 800CW-conjugated anti-goat or IRDye 680RD-conjugated anti-mouse antibodies (1:10,000 in TBS-N, LI-COR, Lincoln, NE, USA). Proteins were visualised using the LI-COR Odyssey Infrared Imaging System (LI-COR). Settings of the fluorescence reading were chosen, so that all signals are in the linear range of detection and that no signals are overexposed. Results were quantified using Image Studio (LI-COR). The results of independent experiments were each normalised to a reference sample as indicated in the figures.

### Confocal laser-scanning microscopy

MDA-MB-468 cells were seeded on poly-L-lysine-coated glass cover slips and cultivated for 24 h. Subsequently, cells were transiently transfected with pd2eGFP-N1 expression vectors encoding for murine wild type or mutant C-terminally GFP-tagged Gab1. After 24 h, cover slips were placed into the pre-heated (37 °C) incubation chamber of the laser scanning microscope and equilibrated for 30 min. Where indicated, cells were treated with inhibitors or DMSO. Imaging was performed with a confocal laser scanning microscope (LSM700, Zeiss, Jena, Germany). Prior to usage, the temperature of the incubation chamber (PeCon, Erbach, Germany) and of the objective lens was adjusted to 37 °C and the atmosphere within the incubation chamber was set to 5% CO_2_. eGFP-Gab1 fusion proteins were excited using laser light of 488 nm. Emission was detected in the range of 493 to 700 nm.

### Quantitative RT-PCR

Total RNA was isolated using the Universal RNA Purification Kit (Roboklon, Berlin, Germany) according to the manufacturer’s instructions. 500 ng of RNA were reverse transcribed into cDNA with NG dART RT Kit (Roboklon), employing random hexameric primers according to the manufacturer’s instructions. mRNA expression of EGR1, cyclin D1, SDHA, GAPDH, and HPRT1 was analysed with primers for human EGR1 (fw: 5’ AGCAGCACCTTCAACCCTCAGG 3’, rev: 5’ GAGTGGTTTGGCTGGGGTAACT 3’), cyclin D1 (fw: 5’ GCTGTGCATCTACACCGACA 3’, rev: 5’ TTGAGCTTGTTCACCAGGAG 3’), SDHA (fw: 5’ TGGGAACAAGAGGGCATCTG 3’, rev: 5’ CCACCACTGCATCAAATTCATG 3’), HPRT1 (fw: 5’ TGACACTGGCAAAACAATGCA 3’, rev: 5’ GGTCCTTTTCACCAGCAAGCT 3’) and GAPDH (fw: 5’ TGATGACATCAAGAAGGTGG 3’, rev: 5’ TTACTCCTTGGAGGCCATGT 3’). SDHA, HPRT1 and GAPDH served as house-keeping genes. PCR was performed using PowerTrack SYBR Green qPCR Master Mix (Thermo Fisher Scientific) according to the manufacturer’s instructions. The PCR reaction was done in a final volume of 20 µL containing 2 µL cDNA. The cDNA was denatured for 10 min at 95 °C. After denaturing amplification was performed in 40 cycles (15 s at 95 °C, 30 s at 60 °C, 30 s at 72 °C) in a Rotorgene (Qiagen, Hilden, Germany). Quantification of gene expression was calculated as described by Pfaffl et al. [35] using multiple house-keeping genes.

### Statistical analysis

Results are presented as means ± SD of independent experiments. Statistical analysis was performed with GraphPad Prism 9 (GraphPad Software, Boston, MA, USA) using Student´s t-test (for single comparison) or ordinary one-way ANOVA with Turkey´s multiple comparisons test (for multiple comparisons) as indicated in the figures. The designations n.s. = non-significant, * = *p* < 0.05, ** = *p* < 0.01, *** = *p* < 0.001 denote *p* values for the measured differences.

## Results

### Heterogenous Gab1 expression and Gab1 Y627 phosphorylation in breast cancer cell lines

**Fig. 1:**
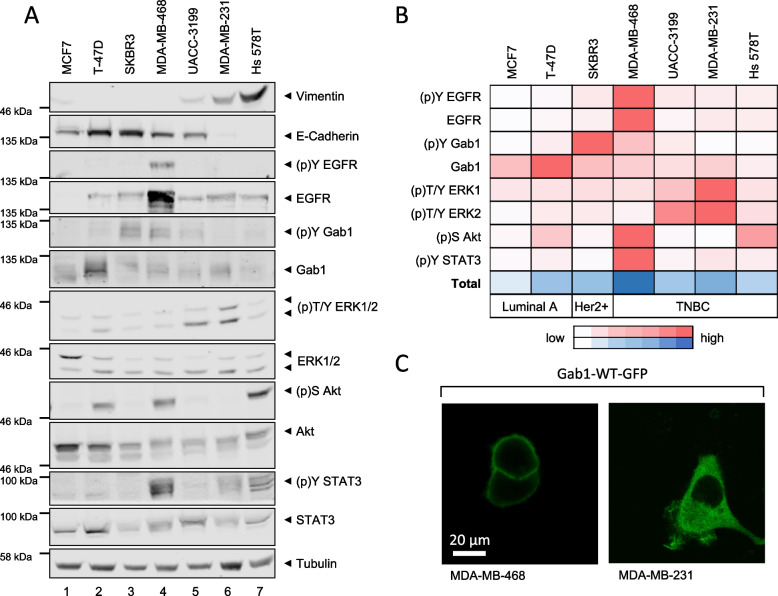
Constitutive signalling in breast cancer cell lines. **A** MCF7, T-47D, SKBR3, MDA-MB-468, UACC-3199, MDA-MB-231 and Hs 578T cells were seeded and cultivated in phenol red-free RPMI 1640 for 24 h. On the following day, cells were lysed and proteins were separated by SDS-PAGE. After Western blotting, membranes were stained with specific antibodies against vimentin, E-cadherin, (p)Y EGFR, EGFR, (p)Y Gab1, Gab1, (p)T/Y ERK1/2, ERK1/2, (p)S Akt, Akt, (p)Y STAT3, STAT3 and tubulin. A representative result of *n* = 3 independent experiments is shown. **B** Expression or phosphorylation of (p)Y EGFR, EGFR, (p)Y Gab1, Gab1, (p)T/Y ERK1/2, (p)S Akt and (p)Y STAT3 from *n* = 3 independent experiments were quantified and normalised to tubulin expression. The highest phosphorylation or expression among the cell lines analysed was set to 100%. High expression or phosphorylation is visualised in dark red. For each cell type the normalised phosphorylation and expression strengths of all analysed entities are summed up and depicted in blue. **C** MDA-MB-468 or MDA-MB-231 cells were seeded on poly-L-lysine-coated glass cover slips and cultivated in phenol red-free RPMI 1640. After 24 h, cells were transfected with an expression vector for murine Gab1-GFP. On the following day, cells were placed into the incubation chamber of a laser scanning microscope. Imaging was performed after 30 min equilibration. Representative results of *n* = 3 independent experiments are shown

To monitor differences in constitutive signalling in different BC cell lines, phosphorylation of Y1068 in EGFR, Y627 in Gab1, T202/Y204 in ERK1/2, S473 in Akt, Y705 in STAT3 as well as expression of EGFR, Gab1, ERK1/2, Akt and STAT3 were determined by Western blot analyses applied to seven BC cell lines. Phosphorylated Y627 within human Gab1 is the binding site for SHP2 and thus represents the function of Gab1 as adaptor protein [36]. Expression of vimentin and E-cadherin was analysed as an indicator for epithelial-mesenchymal transition (EMT) of the different cell lines, while expression of tubulin serves as loading control. We utilised the luminal BC cell lines MCF7 and T-47D, the Her2-positive BC cell line SKBR3, and the four TNBC cell lines MDA-MB-468, UACC-3199, MDA-MB-231 and Hs 578T (for review, see [37]). While the luminal BC cells (lanes 1 and 2), the Her2-positive BC cells (lane 3) and the TNBC cell lines MDA-MB-468 (lane 4) and UACC-3199 (lane 5) express high levels of E-cadherin and little to no vimentin, the two TNBC cell lines MDA-MB-231 (lane 6) and Hs 578T (lane 7) preferentially express vimentin and less E-cadherin (Fig. 1A). This indicates EMT of the latter two TNBC cell lines and strong heterogeneity even between TNBC cell lines. Notably, the expression and phosphorylation strength of EGFR, Gab1, ERK1/2, Akt and STAT3 is also very heterogeneous among the different BC cell lines and does not correlate with disease stage or EMT (Fig. 1A). For example, while Akt is strongly activated in MDA-MB-468 cells, it is not activated in MDA-MB-231 cells. In contrast, ERK1/2 is strongly phosphorylated in MDA-MB-231 cells but not in MDA-MB-468 cells. This underlines that the understanding of dysregulated growth-promoting signalling pathways in BC cannot be generalised for different subgroups or ultimately different patient groups, but must be investigated for each individual molecular background. To find a subjective measure for dysregulated signalling we quantified expression of Gab1 and EGFR and phosphorylation of EGFR, Gab1, ERK1, ERK2, Akt and STAT3 (Fig. 1B). For each analysed entity, the highest phosphorylation or expression among the cell lines analysed was normalised to 100%. High expression or phosphorylation is visualised in dark red. Next, for each cell type the normalised phosphorylation and expression strengths of all analysed entities were summed up (Fig. 1B, total). Here, dark blue represents the highest sum of expression of Gab1 and EGFR and phosphorylation of EGFR, Gab1, ERK1, ERK2, Akt and STAT3. Based on this measure, MDA-MB-468 and MDA-MB-231 cells exhibit the strongest constitutive activation and expression of the analysed signalling pathways, among the analysed TNBC cells. Notably, Gab1 is constitutively phosphorylated at Y627 in MDA-MB-468 cells, while it is only weakly phosphorylated in MDA-MB-231 cells (Fig. 1A and B).

Gab1 function is tightly regulated by its localisation within the cell [32]. Thus, we next examined localisation of Gab1 in MDA-MB-468 and MDA-MB-231 cells. Gab1-GFP was expressed in MDA-MB-468 and MDA-MB-231 cells and its cellular distribution was analysed by confocal microscopy (Fig. 1C). In MDA-MB-468 cells, Gab1 is not only constitutively phosphorylated at Y627 (Fig. 1A) but also constitutively located at the plasma membrane. In contrast, Gab1 is distributed in the cytoplasm in MDA-MB-231 cells.

In summary, in the TNBC cell line MDA-MB-468, EGFR is overexpressed and constitutively activated. Additionally, the Gab1/MAPK/PI3K signalling network is highly active and dysregulated. We thus focussed on decoding the Gab1/MAPK/PI3K network in MDA-MB-468 cells.

### Constitutive Gab1 tyrosine phosphorylation and Gab1 plasma membrane recruitment rely on EGFR activity

**Fig. 2:**
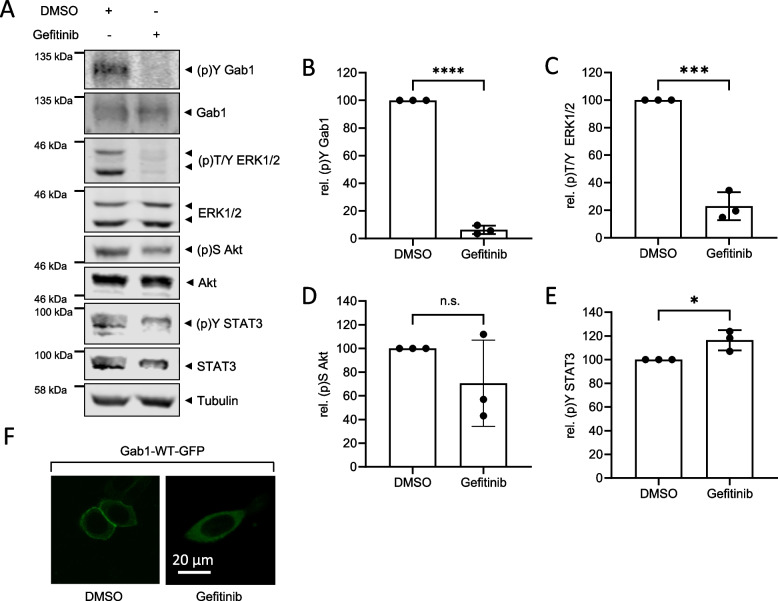
EGFR activity promotes constitutive Gab1 and MAPK phosphorylation in MDA-MB-468 cells. **A** MDA-MB-468 cells were seeded and cultivated in phenol red-free RPMI 1640 for 24 h. On the following day, cells were treated with DMSO or Gefitinib (3 µM) for 30 min. Subsequently, cells were lysed and proteins were separated by SDS-PAGE. After Western blotting, membranes were stained with specific antibodies against (p)Y Gab1, Gab1, (p)T/Y ERK1/2, ERK1/2, (p)S Akt, Akt, (p)Y STAT3, STAT3 and tubulin. A representative result of *n* = 3 independent experiments is shown. The results from (**A**) were quantified. The diagrams show the ratios of **B** (p)Y Gab1 to Gab1, **C** (p)T/Y ERK1/2 to ERK1/2, **D** (p)S Akt to Akt, and **E** (p)Y STAT3 to STAT3. Gab1, ERK1/2, Akt and STAT3 phosphorylation in DMSO-treated cells was normalised to 100% in each independent repetition of the experiment. Data are given as mean of three independent experiments ± SD. Student’s t-test: n.s. = non-significant, * = *p* < 0.05, ** = *p* < 0.01, *** = *p* < 0.001. **F** MDA-MB-468 cells were seeded on poly-L-lysine-coated glass cover slips and cultivated in phenol red-free RPMI 1640. After 24 h, cells were transfected with an expression vector for murine Gab1-GFP. On the following day, cells were placed into the incubation chamber of a laser scanning microscope and left for 30 min. Cells were treated with Gefitinib (3 µM) or DMSO for 30 min. Imaging was performed after treatment with Gefitinib or DMSO. Representative results of *n* = 3 independent experiments are shown

Because the Gab1/MAPK/PI3K signalling network is most strongly activated among the analysed BC cell lines in MDA-MB-468 cells, we now focused on understanding the molecular mechanisms of activation of this network in MDA-MB-468 cells. In MDA-MB-468 cells the gene coding for EGFR is amplified [38], which results in strong overexpression and constitutive activation of the EGFR (Fig. 1A). Further, EGFR signalling was shown to facilitate Gab1 phosphorylation in non-BC cells [39]. To test whether constitutive activation of EGFR is also involved in Gab1 tyrosine phosphorylation and activation of the MAPK and PI3K pathways in MDA-MB-468 cells, cells were incubated with the EGFR inhibitor Gefitinib for 30 min. Subsequently, phosphorylation and expression of Gab1, ERK1/2, Akt and STAT3 were analysed by Western blotting. Staining of tubulin serves as loading control (Fig. 2A). Short-term inhibition of EGFR abolishes constitutive phosphorylation of Gab1 (Fig. 2A and B) and ERK1/2 (Fig. 2A and C), while constitutive Akt (Fig. 2A and D) and STAT3 (Fig. 2A and E) phosphorylation is not significantly affected. Notably, MDA-MB-468 cells harbour an oncogenic PTEN mutation that reduces PTEN activity [20]. Reduced PTEN activity might counteract deactivation of the PI3K pathway upon inhibition of EGFR.

As Gab1 is no longer phosphorylated upon short-term inhibition of EGFR, we next analysed whether reduced Gab1 phosphorylation goes along with reduced membrane localisation. MDA-MB-468 cells were transiently transfected with Gab1-GFP and its localisation upon a 30 min treatment with either DMSO or Gefitinib was analysed by confocal microscopy (Fig. 2F). In DMSO-treated control MDA-MB-468 cells Gab1 is localised at the plasma membrane. In contrast, short-term inhibition of EGFR releases Gab1 from the membrane and results in cytoplasmic distribution of Gab1.

In summary, these results indicate that in MDA-MB-468 cells Gab1 and ERK1/2 phosphorylation, as well as constitutive plasma membrane recruitment of Gab1 rely on EGFR activity, whereas constitutive STAT3 phosphorylation is probably mediated by a different upstream kinase.

### Inhibition of EGFR activity reduces migration

**Fig. 3:**
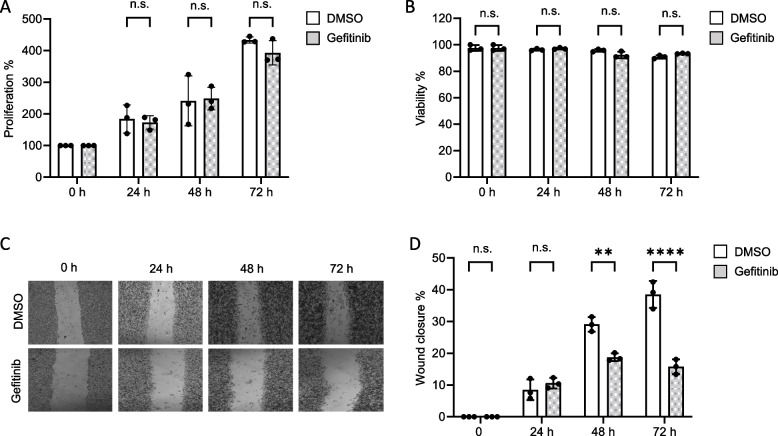
EGFR activity promotes migration of MDA-MB-468 cells. MDA-MB-468 cells were seeded on 6-well plates and cultivated in phenol red-free RPMI 1640. On the following day, cells were treated with DMSO or Gefitinib (3 µM). **A** After the time indicated, the total cell number was determined and the cell number at day 0 was normalised to 100%. **B** The percentage of viable cells was determined at the indicated time points. **C** MDA-MB-468 cells were seeded on 24-well plates and cultivated in phenol red-free RPMI 1640. After 24 h, the cells were subjected to a wound healing assay upon DMSO or Gefitinib (3 µM) treatment for 72 h. Representative results of *n* = 3 independent experiments are shown. **D** Wound closure is given in % of initial wound area. Data are given as mean of *n* = 3 independent experiments ± SD. One-way ANOVA: n.s. = non-significant, * = *p* < 0.05, ** = *p* < 0.01, *** = *p* < 0.001

Next, we asked whether EGFR inhibition affects proliferation, viability and migration of MDA-MB-468 cells. Hence, we treated MDA-MB-468 cells with the EGFR inhibitor Gefitinib for up to 72 h. Neither proliferation (Fig. 3A) nor viability (Fig. 3B) of MDA-MB-468 cells was affected by this treatment. Next, migration of MDA-MB-468 cells upon EGFR inhibition was analysed with a wound healing assay. Inhibition of EGFR significantly reduces the ability of MDA-MB-468 cells to migrate compared to DMSO-treated cells (Fig. 3C and D).

Taken together, EGFR activity is crucial for constitutive Gab1 and ERK1/2 signalling. This is accompanied by reduced migration of MDA-MB-468 cells upon EGFR inhibition, whereas proliferation and cell survival remain unaffected.

### Constitutive Gab1 tyrosine phosphorylation relies on MAPK and PI3K activity

Regulation of Gab1 depends on MAPK and PI3K signalling (for review, see [21, 22]). To investigate the specific roles of the MAPK and PI3K pathways on Gab1 tyrosine 627 phosphorylation, the two pathways were inhibited individually in MDA-MB-468 cells. To do so, the cells were either treated with the MEK inhibitor U0126 (U0) or the PI3K inhibitor LY294002 (LY) for 30 min. Additionally, the two inhibitors were applied together to uncover potential additive action of the two pathways on Gab1 phosphorylation. Subsequently, phosphorylation and expression of Gab1, ERK1/2, Akt and STAT3 were analysed by Western blotting.

**Fig. 4:**
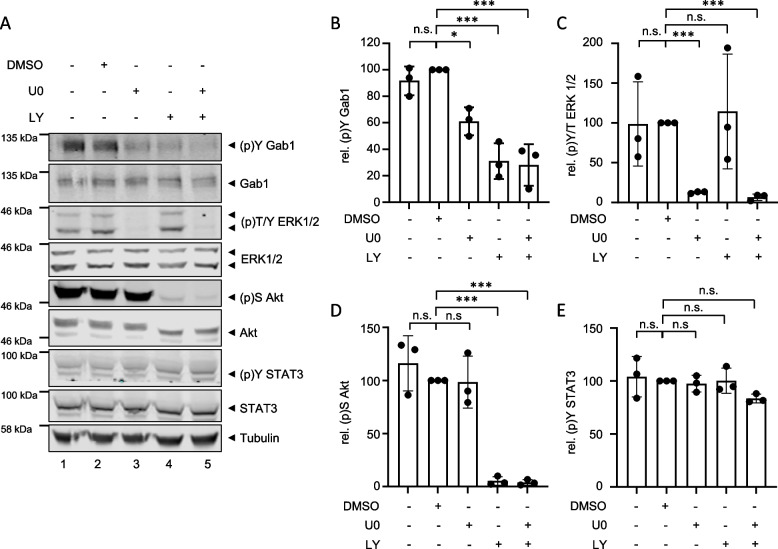
MAPK and PI3K activity promote constitutive Gab1 phosphorylation in MDA-MB-468 cells. **A** MDA-MB-468 cells were cultivated in phenol red-free RPMI 1640 for 24 h. On the following day, cells were treated with DMSO, U0126 (10 µM) and/or LY294002 (40 µM) for 30 min. Subsequently, cells were lysed and proteins were separated by SDS-PAGE. After Western blotting, membranes were stained with specific antibodies against (p)Y Gab1, Gab1, (p)T/Y ERK1/2, ERK1/2, (p)S Akt, Akt, (p)Y STAT3, STAT3 and tubulin. A representative result of *n* = 3 independent experiments is shown. The results from (**A**) were quantified. The diagrams show the ratios of **B** (p)Y Gab1 to Gab1, **C** (p)T/Y ERK1/2 to ERK1/2, **D** (p)S Akt to Akt, and **E** (p)Y STAT3 to STAT3. Gab1, ERK1/2, Akt and STAT3 phosphorylation in DMSO-treated cells was normalised to 100% in each independent repetition of the experiment. Data are given as mean of three independent experiments ± SD. One-way ANOVA: n.s. = non-significant, * = *p* < 0.05, ** = *p* < 0.01, *** = *p* < 0.001

Treatment of MDA-MB-468 cells with the MEK inhibitor U0 blocks phosphorylation of ERK1/2 (Fig. 4A, lane 3 and C) without reducing Akt phosphorylation (Fig. 4A, lane 3 and D). Of note, inhibition of MEK reduces Gab1 phosphorylation (Fig. 4A, lane 3 and B) indicating that activation of the MAPK pathway is necessary for Gab1 phosphorylation in MDA-MB-468 cells. Inhibition of PI3K signalling by LY reduces Akt phosphorylation (Fig. 4A, lane 4 and D) without reducing ERK1/2 phosphorylation (Fig. 4A, lane 4 and C). Interestingly, inhibition of the PI3K pathway also blocks Gab1 phosphorylation (Fig. 4A, lane 4 and B). Blockade of both MAPK and PI3K signalling together blocks ERK1/2, Akt and Gab1 phosphorylation with no additive effects (Fig. 4A, lane 5 and B). In contrast to phosphorylation of ERK1/2, Akt and Gab1, STAT3 phosphorylation is not affected by inhibition of the MAPK and PI3K pathways (Fig. 4A and E), indicating that STAT3 is not involved in the Gab1/MAPK/PI3K signalling network.

In summary, both, MAPK and PI3K pathway activation, contribute to constitutive Gab1 tyrosine 627 phosphorylation in MDA-MB-468 cells. Thus, we tested next, whether these two pathways also both contribute to constitutive plasma membrane localisation of Gab1.

### Constitutive plasma membrane recruitment of Gab1 relies on MAPK and PI3K signalling

**Fig. 5:**
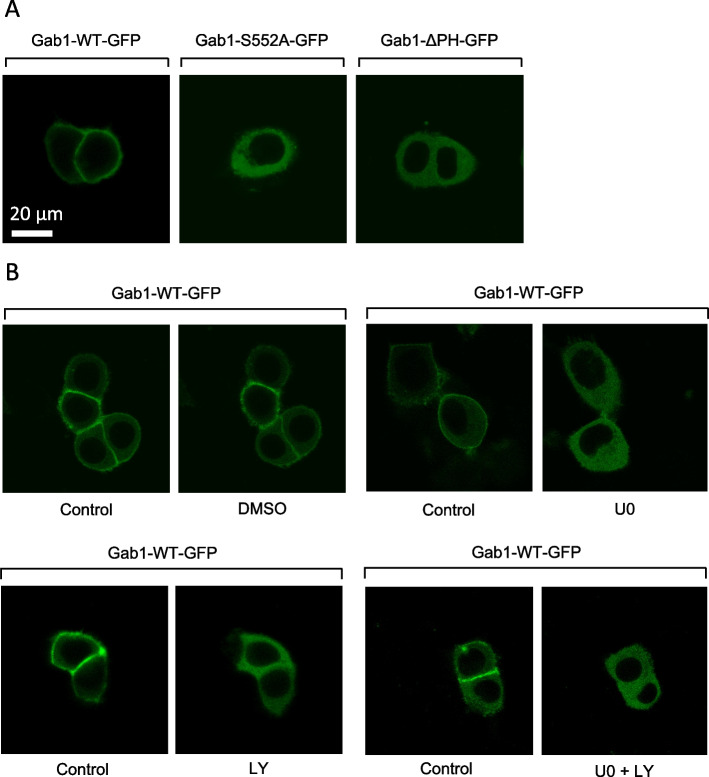
MAPK and PI3K signalling are crucial for Gab1 plasma membrane recruitment in MDA-MB-468 cells. **A** MDA-MB-468 cells were seeded on poly-L-lysine-coated glass cover slips and cultivated in phenol red-free RPMI 1640. After 24 h, cells were transfected with an expression vector for either murine Gab1-GFP, murine Gab1-S552A-GFP or murine Gab1-ΔPH-GFP. On the following day, cells were placed into the incubation chamber of a laser scanning microscope. Imaging was performed after 30 min equilibration. Representative results of *n* = 3 independent experiments are shown. **B** MDA-MB-468 cells were seeded on poly-L-lysine-coated glass cover slips and cultivated in phenol red-free RPMI 1640. After 24 h, cells were transfected with an expression vector for murine Gab1-GFP. On the following day, cells were placed into the incubation chamber of a laser scanning microscope and left for 30 min. Cells were treated with DMSO, U0126 (10 µM) and/or LY294002 (40 µM) for 30 min. Imaging was performed before and after treatment. Representative results of *n* = 3 independent experiments are shown

Under physiological conditions, recruitment of Gab1 to the plasma membrane is initiated by MAPK-dependent phosphorylation of S551 in human Gab1 or S552 in murine Gab1 [32]. This phosphorylation initiates an intramolecular switch that allows interaction of the PH-domain of Gab1 with PIP3 in the plasma membrane [23]. To test whether S551 and the PH-domain are also involved in constitutive Gab1 plasma membrane recruitment in MDA-MB-468 cells, these cells were transfected either with murine Gab1-GFP or one of two Gab1-GFP mutants. Gab1-S552A-GFP cannot be phosphorylated at position 552. Gab1-ΔPH-GFP lacks the PH-domain. The cellular distribution of the Gab1-GFP variants was analysed by confocal microscopy (Fig. 5A). In contrast to Gab1-GFP, which is constitutively located at the plasma membrane, both Gab1-S552A-GFP and Gab1-ΔPH-GFP are distributed in the cytoplasm. This indicates that both, the PH-domain and serine 551/2 in Gab1, are crucial for constitutive plasma membrane recruitment of Gab1 in MDA-MB-468 TNBC cells.

Previously, we have shown a crucial contribution of MAPK and PI3K signalling in mediating constitutive plasma membrane recruitment of Gab1 in erythroleukemia cells [24]. Hence, we hypothesised that constitutive plasma membrane recruitment of Gab1 in TNBC cells also depends on MAPK and PI3K signalling. Examining this, we treated MDA-MB-468 cells expressing Gab1-WT-GFP with the MEK inhibitor U0 or the PI3K inhibitor LY for 30 min and analysed the cellular distribution of Gab1 with live cell imaging (Fig. 5B). Treatment of control cells with DMSO does not affect the constitutive recruitment of Gab1 to the plasma membrane. Of note, both MAPK and PI3K inhibition as well as treatment of cells with the two inhibitors together abolish constitutive plasma membrane recruitment of Gab1.

Taken together, MAPK and PI3K pathway activation, the presence of S551/2, and the presence of the PH-domain in Gab1 are prerequisites for constitutive plasma membrane recruitment of Gab1 in MDA-MB-468 TNBC cells.

### Inhibition of MAPK and PI3K reduces migration and proliferation

**Fig. 6:**
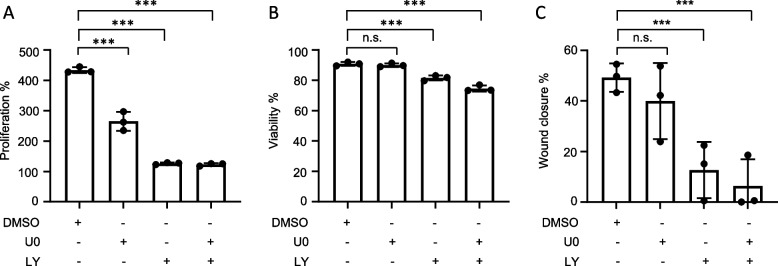
PI3K signalling is crucial for proliferation and migration of MDA-MB-468 cells. MDA-MB-468 cells were seeded on 6-well plates and cultivated in phenol red-free RPMI 1640. On the following day, cells were treated with DMSO, U0126 (10 µM) and/or LY294002 (40 µM) **A** After 72 h the total cell number was determined. Proliferation is depicted as % increase of proliferation compared to d 0. **B** After 72 h, the percentage of viable cells was determined. **C** MDA-MB-468 cells were seeded on 24-well plates and cultivated in phenol red-free RPMI 1640. The next day, the cells were subjected to a wound healing assay upon treatment with DMSO, U0126 (10 µM) and/or LY294002 (40 µM) for 72 h. Wound closure is given in % of initial wound area. Data are given as mean of *n* = 3 independent experiments ± SD. One-way ANOVA: n.s. = non-significant, * = *p* < 0.05, ** = *p* < 0.01, *** = *p* < 0.001

As MAPK and PI3K inhibition reduce Gab1 phosphorylation and plasma membrane localisation, we next asked whether impaired Gab1 signalling correlates with changes in proliferation, viability and migration of MDA-MB-468 cells. Hence, MDA-MB-468 cells were treated with the MEK inhibitor U0, the PI3K inhibitor LY or a combination of these inhibitors for 72 h. Inhibition of MAPK signalling with U0 reduces proliferation of MDA-MB-468 cells by 50%, while inhibition of PI3K signalling with LY and combined inhibition of both pathways with U0 and LY reduces proliferation by 75% (Fig. 6A). Beside the fact that inhibition of MAPK and PI3K both reduce cell proliferation, only inhibition of PI3K decreases viability slightly (Fig. 6B). Furthermore, migration of MDA-MB-468 cells is strongly reduced in the presence of the PI3K inhibitor (Fig. 6C).

These results show that MAPK and PI3K signalling are crucial for cancer-associated proliferation of MDA-MB-468 TNBC cells. Furthermore, PI3K signalling is essential for survival and migration.

### Restored MAPK and Gab1 phosphorylation upon long-term inhibition of MAPK signalling

Despite initial optimism, many clinical trials with drugs targeting EGFR, MAPK and PI3K signalling in TNBC have been unsuccessful at improving patient outcomes. One reason for this is the development of resistance (for review, see [17, 19, 40]). Recent evidence suggests that fundamental changes in cancer-associated signalling take place during cancer progression. These changes may also contribute to the acquisition of resistance (for review, see [40]). Therefore, we aimed to investigate, whether the Gab1/MAPK/PI3K signalling network is changed after long-term treatment with inhibitors.

**Fig. 7:**
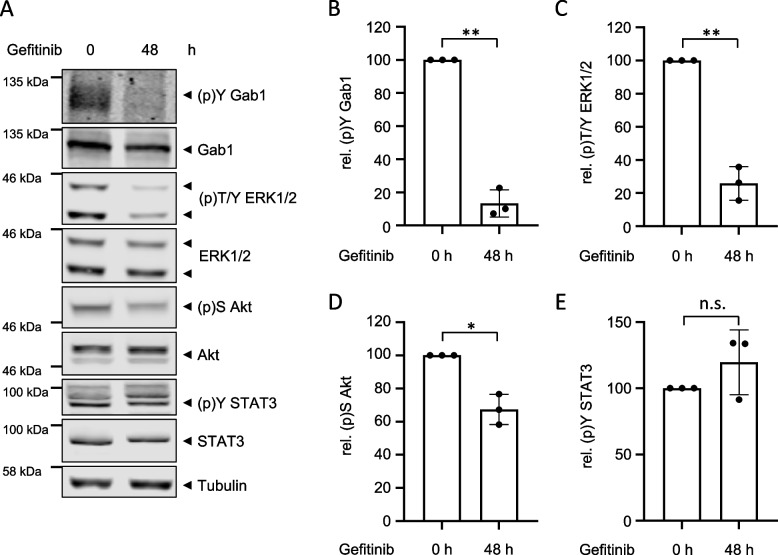
MDA-MB-468 cells do not acquire resistance to EGFR inhibition. **A** MDA-MB-468 cells were seeded and cultivated in phenol red-free RPMI 1640 for 24 h. On the following day, cells were treated with Gefitinib (3 µM) for 48 h. Control cells were treated with DMSO. Subsequently, cells were lysed and proteins were separated by SDS-PAGE. After Western blotting, membranes were stained with specific antibodies against (p)Y Gab1, Gab1, (p)T/Y ERK1/2, ERK1/2, (p)S Akt, Akt, (p)Y STAT3, STAT3 and tubulin. A representative result of *n* = 3 independent experiments is shown. The results from (**A**) were quantified. The diagrams show the ratios of **B** (p)Y Gab1 to Gab1, **C** (p)T/Y ERK1/2 to ERK1/2, **D** (p)S Akt to Akt, and **E** (p)Y STAT3 to STAT3. Gab1, ERK1/2, Akt and STAT3 phosphorylation in DMSO-treated cells was normalised to 100% in each independent repetition of the experiment. Data are given as mean of three independent experiments ± SD. Student’s t-test: n.s. = non-significant, * = *p* < 0.05, ** = *p* < 0.01, *** = *p* < 0.001

First, MDA-MB-468 cells were treated with the EGFR inhibitor Gefitinib for 48 h. Subsequently, phosphorylation and expression of Gab1, ERK1/2, PI3K and STAT3 were analysed by Western blotting (Fig. 7). Long-term inhibition of EGFR, just like short-term inhibition of EGFR (Fig. 2), reduces Gab1 Y627 (Fig. 7A and B) and ERK1/2 phosphorylation (Fig. 7A and C). Additionally, also Akt phosphorylation is reduced by long-term treatment with Gefitinib (Fig. 7A and D), while it is not significantly affected by short-term treatment (Fig. 2). This might be explained by the fact that long-term inhibition of EGFR overcomes the consequences of reduced PTEN activity in MDA-MB-468 cells. STAT3 phosphorylation is not significantly reduced by EGFR inhibition independent of treatment duration (Figs. 2 and 7A and E).

**Fig. 8:**
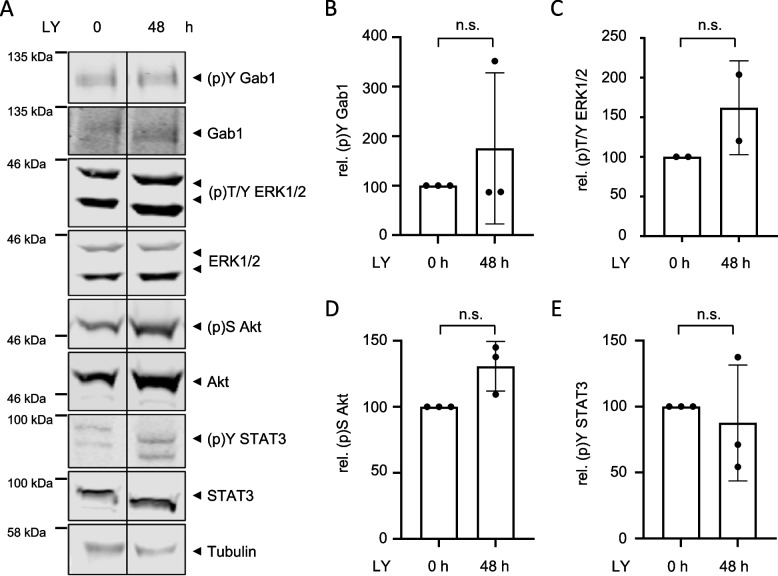
MDA-MB-468 cells acquire resistance to PI3K inhibition. **A** MDA-MB-468 cells were seeded and cultivated in phenol red-free RPMI 1640 for 24 h. On the following day, cells were treated with LY294002 (40 µM) for 48 h. Control cells were treated with DMSO. Subsequently, cells were lysed and proteins were separated by SDS-PAGE. After Western blotting, membranes were stained with specific antibodies against (p)Y Gab1, Gab1, (p)T/Y ERK1/2, ERK1/2, (p)S Akt, Akt, (p)Y STAT3, STAT3 and tubulin. Vertical bars indicate removed lanes on the same blot. A representative result of *n* = 3 independent experiments is shown. The results from (**A**) were quantified. The diagrams show the ratios of **B** (p)Y Gab1 to Gab1, **C** (p)T/Y ERK1/2 to ERK1/2, **D** (p)S Akt to Akt, and **E** (p)Y STAT3 to STAT3. Gab1, ERK1/2, Akt and STAT3 phosphorylation in DMSO-treated cells was normalised to 100% in each independent repetition of the experiment. Data are given as mean of two to three independent experiments ± SD. Student’s t-test: n.s. = non-significant, * *= p* < 0.05, ** = *p* < 0.01, *** = *p* < 0.001

Next, MDA-MB-468 cells were treated with the PI3K inhibitor LY for 48 h. While MDA-MB-468 cells were still sensitive to Gefitinib treatment after 48 h (Fig. 7), they acquire resistance against LY treatment (Fig. 8). Short-term treatment with LY decreases Gab1 and Akt phosphorylation without affecting ERK1/2 phosphorylation (Fig. 4). In contrast, upon long-term treatment with LY, phosphorylation of Gab1 (Fig. 8A and B), ERK1/2 (Fig. 8A and C) and Akt (Fig. 8A and D) are, although not significant, rather slightly increased than reduced.

**Fig. 9:**
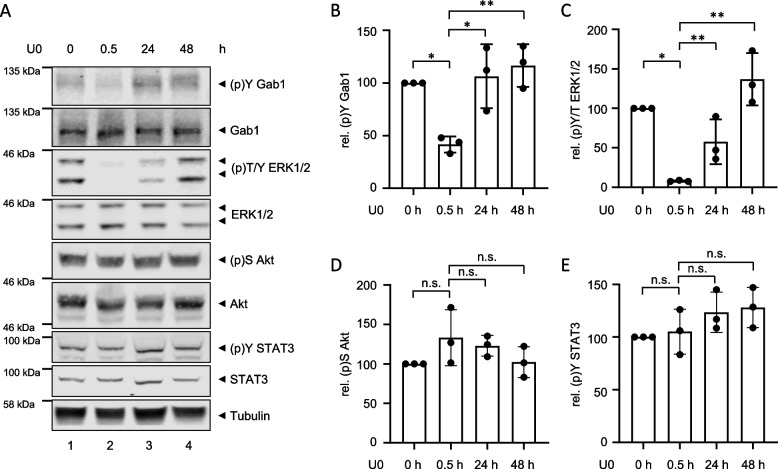
MDA-MB-468 cells acquire resistance to MAPK inhibition. **A** MDA-MB-468 cells were seeded and cultivated in phenol red-free RPMI 1640 for 24 h. On the following day, cells were treated with U0126 (10 µM) for the indicated times. Subsequently, cells were lysed and proteins were separated by SDS-PAGE. After Western blotting, membranes were stained with specific antibodies against (p)Y Gab1, Gab1, (p)T/Y ERK1/2, ERK1/2, (p)S Akt, Akt, (p)Y STAT3, STAT3 and tubulin. A representative result of *n* = 3 independent experiments is shown. The results from (**A**) were quantified. The diagrams show the ratios of **B** (p)Y Gab1 to Gab1, **C** (p)T/Y ERK1/2 to ERK1/2, **D** (p)S Akt to Akt, and **E** (p)Y STAT3 to STAT3. Gab1, ERK1/2, Akt and STAT3 phosphorylation in untreated cells was normalised to 100% in each independent repetition of the experiment. Data are given as mean of three independent experiments ± SD. One-way ANOVA: n.s. = non-significant, * = *p* < 0.05, ** = *p* < 0.01, *** = *p* < 0.001

Finally, MDA-MB-468 cells were treated with the MEK inhibitor U0 for up to 48 h. As shown before (Fig. 4), short-term treatment of MDA-MB-468 cells with U0 abolishes ERK1/2 phosphorylation (Fig. 9A, lane 2 and C) accompanied with reduced Gab1 phosphorylation (Fig. 9A, lane 2 and B). However, ERK1/2 and Gab1 phosphorylation are restored upon long-term U0 treatment for 24 h (Fig. 9A, lane 3) and 48 h (Fig. 9A, lane 4). Akt and STAT3 phosphorylation are not affected by U0 treatment for all times analysed (Fig. 9A, D and E).

In summary, long-term inhibition of PI3K and MAPK signalling results in acquired resistance towards MAPK and PI3K pathway inhibition.

### The Gab1/MAPK/PI3K signalling network regulates gene expression in a target gene-specific manner

**Fig. 10:**
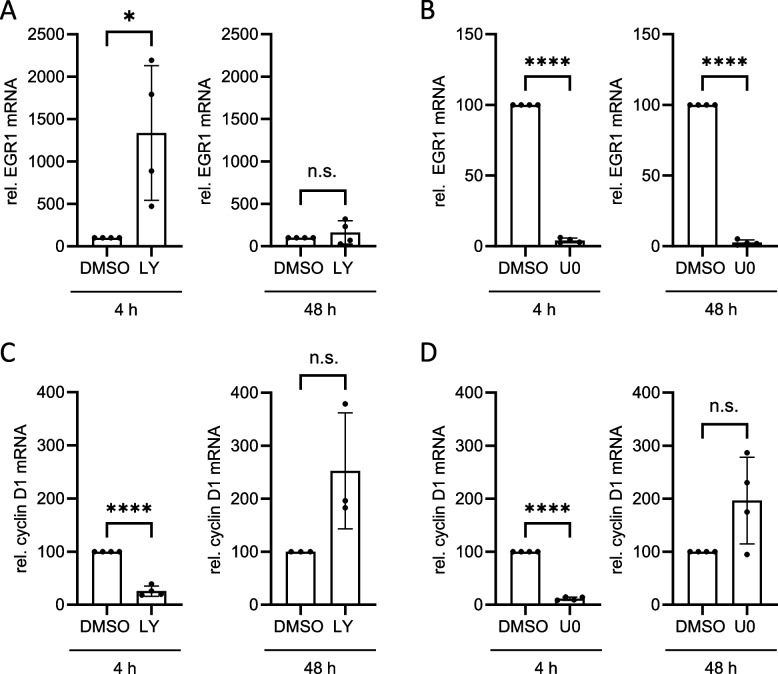
Inhibition of PI3K and MAPK affects mRNA expression in a gene-specific manner. MDA-MB-468 cells were seeded and cultivated in phenol red-free RPMI 1640 for 24 h. On the following day, cells were treated with DMSO, U0126 (10 µM) or LY294002 (40 µM) for the indicated times. mRNA was isolated and transcribed into cDNA. EGR1 mRNA (**A**, **B**) and cyclin D1 mRNA (**C**, **D**) were quantified by qRT-PCR. Expression of mRNA in DMSO-treated cells was set to 100%. Data are given as mean of *n* = 3–4 independent experiments ± SD. Student’s t-test: n.s. = non-significant, * = *p* < 0.05, ** = *p* < 0.01, *** = *p* < 0.001

Intracellular signalling pathways converge at the level of gene expression. In a complex interconnected network, the activation and inactivation of transcription factors and the subsequent binding to or competition for regulatory DNA sequences control the transcription of target genes that ultimately regulate cellular functions. Thus, we next addressed whether the expression of EGFR target genes reflects the acquired resistance towards PI3K and MAPK inhibition. MDA-MB-468 cells were incubated with LY or U0 for either 4 or 48 h to analyse the expression of target genes before the cells develop resistance to inhibition or at a time when they have already developed resistance to PI3K and MAPK inhibition. Treatment with DMSO instead of inhibitors served as control. mRNA expression of the transcription factor early growth response protein 1 (EGR1) and of the cell cycle regulator cyclin D1 were analysed. Inhibition of PI3K by LY induces EGR1 expression after short-term treatment, but not after long-term treatment (Fig. 10A). This mimics the acquired resistance after long-term treatment with LY (Fig. 8). Surprisingly, both short-term and long-term treatment of MDA-MB-468 cells with the MAPK inhibitor U0 result in downregulation of EGR1 mRNA (Fig. 10B), which does not resemble the acquired resistance of signalling after long-term treatment with U0 (Fig. 9). Cyclin D1 mRNA is reduced by short-term inhibition of PI3K (Fig. 10C) and MAPK (Fig. 10D). Notably, this inhibition is lost after long-term inhibition with both inhibitors. In essence, this highlights a gene-specific modification of target gene expression following the acquisition of resistance, emphasizing the presence of unidentified gene-specific effects that contribute to shaping the transcriptional response to pharmacological inhibitors.

### Gab1 is located in the cytoplasm in TNBC cells resistant to PI3K and MAPK inhibitors

**Fig. 11:**
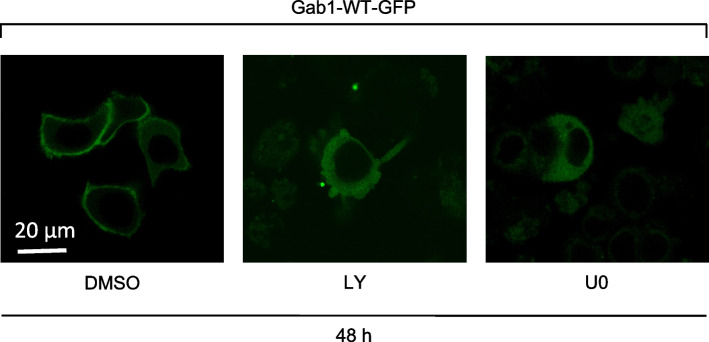
Gab1 is localised in the cytoplasm in PI3K and MAPK inhibition resistant MDA-MB-468 cells. MDA-MB-468 cells were seeded on poly-L-lysine-coated glass cover slips and cultivated in phenol red-free RPMI 1640. After 24 h, cells were transfected with an expression vector for murine Gab1-GFP. On the following day, cells were treated with DMSO, U0126 (10 µM) or LY294002 (40 µM). After 48 h cells were placed into the incubation chamber of a laser scanning microscope. Imaging was performed after 30 min equilibration. Representative results of *n* = 3 independent experiments are shown

Short-term inhibition of PI3K and MAPK signalling releases Gab1 from the membrane (Fig. 5). As MDA-MB-468 cells acquire resistance to LY and U0 treatment (Figs. 8 and 9), we hypothesised that also the cellular distribution of Gab1 is altered upon long-term inhibition. We thus expressed Gab1-GFP in MDA-MB-468 cells and treated those cells for 48 h with either LY or U0. Treatment with DMSO served as control. Cellular localisation of Gab1 was analysed by confocal microscopy (Fig. 11). In DMSO-treated control cells, Gab1 is constitutively located at the plasma membrane. However, upon long-term treatment with either LY or U0 Gab1 is located in the cytoplasm, although it is strongly tyrosine phosphorylated under these conditions (Figs. 8 and 9). These observations suggest that the correlation between Gab1 phosphorylation and Gab1 plasma membrane recruitment is lost in MDA-MB-468 TNBC cells with acquired resistance towards PI3K and MAPK inhibition.

In summary, our data support the hypothesis that fundamental changes in Gab1/MAPK/PI3K signalling take place during cancer progression and resistance development in MDA-MB-468 EGFR^high^ TNBC cells. Gab1 seems to be a central hub in this deregulated signalling network, which is underpinned by the decoupling of Gab1 phosphorylation and localisation in cells with acquired resistance.

## Discussion

Aberrant MAPK and PI3K signalling is a hallmark in several cancers (for review, see [41]). Previous own studies revealed that the multi-site docking protein Gab1 enhances MAPK signalling and thereby increases proliferation of malignant cells [24, 42]. Moreover, the fact that Gab1 expression is enhanced in BC tissues compared to benign mammary hyperplastic tissues [28] let us speculate that the Gab1/MAPK/PI3K signalling network is dysregulated and involved in malignant transformation of BC cells.

BC is a highly heterogeneous disease. This heterogeneity is also manifested at the level of constitutive signalling (Fig. 1). By evaluating Gab1, MAPK and PI3K signalling in different breast cancer cell lines, we observed constitutive ERK1/2 phosphorylation in all breast cancer cell lines examined. Interestingly, constitutive Gab1 phosphorylation is observed in only some of these cell lines. This indicates for a cell-type specific Gab1-independent phosphorylation of ERK1/2 and thus uncoupled Gab1 and ERK1/2 signalling, in some of these BC cell lines. Nonetheless, in the cell lines SKBR3, MDA-MB-468 and UACC-3199 both, Gab1 and ERK1/2, are phosphorylated, which implies for an interrelation of Gab1 and ERK1/2 signalling in these BC cell lines. Remarkably, only in MDA-MB-468 cells phosphorylation of Gab1 correlates clearly with phosphorylation of Akt (Fig. 1). Altogether, this highlights the heterogeneity of BC cell subtypes and calls for in depth analysis of the mechanisms of pathological signalling in individual BC subtypes. Notably, in MDA-MB-468 cells, Gab1 is not only constitutively phosphorylated at tyrosine 627, but also constitutively located at the plasma membrane (Fig. 1C), indicating for dysregulated Gab1 signalling in these specific TNBC cells. The essential role of Gab1 in cancer cell function is highlighted by the fact, that knock-out or knock-down of Gab1 in several cancer types such as erythroleukemia [24], cholangiocarcinoma [43] or oral squamous carcinoma [44] impairs cell proliferation and viability. In accordance with the hypothesis that Gab1 function and signalling are crucial in survival of cancer cells, it was not possible to isolate viable Gab1-deficient MDA-MB-468 cells after CRISPR/Cas9-mediated knock-out of Gab1 (data not shown) using a CRISPR/Cas9 Gab1 knock-out construct that previously allowed generation of Gab1-deficient non-cancerous cells [42].

To further explore the Gab1/MAPK/PI3K signalling network in a specific BC type and its contribution to breast cancer progression, we focused on the analysis of the MDA-MB-468 cell line. This cell line has a basal phenotype, characterised by advanced EMT, thus increased motility and is categorised as TNBC lacking ER, PR and Her2 expression. Moreover, MDA-MB-468 cells are characterised by EGFR overexpression and constitutive EGFR activity (Fig. 1A and B) [45]. Yamasaki et al. showed that Gab1 is required for sufficient EGFR dependent ERK1/2 phosphorylation [25] indicating for a strong interrelation of Gab1 and ERK1/2 signalling in TNBC. Here, we show that constitutive ERK1/2 and Gab1 phosphorylation in MDA-MB-468 cells depend on EGFR activity (Fig. 2). Moreover, constitutive Gab1 plasma membrane recruitment in MDA-MB-468 cells depends on EGFR activity (Fig. 2F). Constitutive PI3K signalling in MDA-MB-468 cells, however, cannot be blocked by short-term EGFR inhibition (Fig. 2). This might be explained by the fact that MDA-MB-468 cells harbour an oncogenic PTEN mutation [20]. Long-term inhibition of EGFR signalling, however, reduces phosphorylation of Akt in MDA-MB-468 cells (Fig. 7).

In response to canonical physiological EGF-induced signalling, Gab1 is recruited to the plasma membrane by direct interaction of its Met binding domain (MBD) with phosphorylated tyrosine motifs within the intracellular part of the EGFR and by binding of its PH domain to PIP3 at the membrane. Notably, the PH domain and thus probably binding to PIP3 is required for efficient tyrosine phosphorylation of Gab1 upon EGF stimulation [39]. Further, Gab1 plasma membrane recruitment is facilitated after MAPK-dependent phosphorylation of murine Gab1 at S552 or human Gab1 at S551 enabling the interaction of the PH domain with PIP3 [23, 32]. This canonical view is in accordance with the signalling initiated by constitutive EGFR in naive MDA-MB-468 cells as described here. As MDA-MB-468 cells harbour an inactivating PTEN mutation, PIP3 is abundantly present at the plasma membrane [20, 46]. Constitutive EGFR activity induces MAPK signalling (Fig. 2) that probably initiates Gab1 phosphorylation at S551/552. The activation of this intramolecular switch enables constitutive plasma membrane localisation of Gab1 via its PH domain (Fig. 5A). Reduction of PIP3 at the membrane by blocking PI3K (Fig. 5) and inhibition of MAPK activity (Fig. 5) releases Gab1 from the membrane and results in reduced Gab1 tyrosine phosphorylation (Fig. 4). Hence, MAPK and PI3K signalling contribute indirectly to Gab1 Y627 phosphorylation (Fig. 4). Notably, resistant MDA-MB-468 cells no longer follow this canonical model. Instead, Gab1 is not located at the plasma membrane despite strong activation of PI3K and MAPK. Furthermore, Gab1 phosphorylation is uncoupled from recruitment to the plasma membrane (Figs. 8, 9 and 11). Thus, signalling in BC is not only affected by the initial mutations causing EGFR overexpression or PTEN inactivation but also by acquired changes in the intertwined signalling networks during therapeutic intervention in the signalling pathways. Notably, the activation state of the MAPK and PI3K pathways does not directly correlate with gene expression (Fig. 10). This indicates that the transcriptional network is fine-tuned e.g. by cross-talk between different signalling pathways in a gene specific manner. Personalised treatments and adaptations to overcome acquired resistance can be effectively developed only if there is a comprehensive understanding of these signalling and transcriptional networks.

In contrast to MAPK and PI3K signalling, constitutive STAT3 phosphorylation is independent of EGFR activity in MDA-MB-468 cells (Fig. 2) suggesting other upstream kinases to activate STAT3. These results are supported by Berishaj et al. who also showed that STAT3 phosphorylation does not depend on EGFR activity in MDA-MB-468 cells. However, inhibition of Janus kinases and blockade of the IL-6-type cytokine receptor gp130 reduces STAT3 phosphorylation in MDA-MB-468 cells [47]. This indicates that IL-6 or other IL-6-type cytokines are involved in constitutive activation of STAT3 in MDA-MD-468 cells.

Inhibition of EGFR activity significantly decreased migration of MDA-MB-468 cells whereas proliferation and survival of these cells are not affected by EGFR inhibition (Fig. 3). The latter results are at odds with Kiyatkin et al. who claim that EGFR signalling is important for cell survival [48]. One explanation for this discrepancy might be that Kiyatkin et al. obtained their results from HEK293 and A431 cells which likely have different cell survival mechanisms than breast cancer cells. The latter might have developed mechanisms allowing for EGFR-independent cell survival. Nonetheless, attenuated migration of EGFR inhibitor-treated MDA-MB-468 cells is in line with reduced migration of Gefitinib-treated MDA-MB-231 cells [49]. Additionally, Gefitinib not only decreases the motility of TNBC cells but also reduces migration of cells of other cancer types [50–52]. Taken together, these observations point to the importance of cell type specificity in cellular behaviour and the urge to understand cell specific signalling.

Additionally, we analysed whether blockade of MAPK and PI3K signalling affects TNBC progression and thus determines proliferation, survival, and migration of MDA-MB-468 cells upon inhibition of these pathways. Viability of MDA-MB-468 cells was only reduced by long-term inhibition of PI3K signalling (Fig. 6B), which is also reflected by the irregular cell shape of cells under this conditions (Fig. 11). Furthermore, PI3K inhibition abolishes cell proliferation and migration, whereas MAPK inhibition reduces proliferation but did not significantly affect migration (Fig. 6). Such an anti-proliferative effect of PI3K and MAPK inhibition on several other TNBC cell lines has already been described [53, 54]. However, it is noteworthy that MAPK inhibition in MDA-MB-468 cells results in a less effective blockade of proliferation than PI3K inhibition (Fig. 6A). Moreover, inhibition of PI3K almost abolishes the migration of MDA-MB-468 cells, whereas inhibition of MAPK has no significant effect on the migration of these cells (Fig. 6C). Although several studies have shown that MAPK signalling is necessary for TNBC proliferation and migration [15, 55], its influence on these processes of cancer progression appears to be limited and extremely cell type specific.

## Conclusion

In summary, we show constitutive Gab1 signalling in EGFR^high^ TNBC MDA-MB-468 cells. While the Gab1/MAPK/PI3K signalling network follows canonical Gab1 signalling in naive MDA-MB-468 cells, Gab1 signalling is fundamentally changed in cells that acquired resistance towards MAPK and PI3K inhibition. In these cells, Gab1 is not located at the plasma membrane despite strong activation of PI3K and MAPK. Furthermore, Gab1 tyrosine phosphorylation in resistant cells does not correlate with plasma membrane localization. To our knowledge, this is the first time that Gab1 signalling has been shown to fundamentally change during acquisition of inhibitor resistance. Given the molecular heterogeneity in breast cancer, a more detailed understanding of these dysregulated and aberrant signalling networks is an absolute necessity in order to develop personalised therapies for patients with TNBC.

## Data Availability

The datasets used and/or analysed during the current study are available from the corresponding author on reasonable request.

## Abbreviations

Tubulin: α-tubulin
AEBSF: 4-(2-aminoethyl)-benzolsulfonylfluorid
DMEM: Dulbecco’s modified Eagle’s medium
EGFR: Epidermal growth factor receptor
EMT: Epithelial-mesenchymal transition
ER: Estrogen receptor
EGR1: Early growth response protein 1
ERK: Extracellular signal regulated kinase
FCS: Fetal calf serum
Gab1: Grb2 associated binder 1
Grb2: Growth factor receptor bound protein 2
Her2: Human epidermal growth receptor 2
LY: LY294002
MAPK: Mitogen activated protein kinase
MBD: MET binding domain
PH: Pleckstrin homology
PI3K: Phosphatidyl-inositol-3-kinase
PIP3: Phosphatidyl-inositol (3,4,5)-trisphosphate
PKB/Akt: Protein kinase B
PLC: Phospholipase C
PTEN: Phosphatase and tensin homolog deleted on chromosome 10
PR: Progesterone receptor
RasGAP: Ras-GTPase activating protein
SH2: Src-homology 2 domain
SHP2: Src-homology domain 2 containing phosphatase 2
STAT: Signal transducer and activator of transcription
TNBC: Triple-negative breast cancer
U0: U0126
